# Prospective study of ^99m^Tc‐3PRGD_2_ SPECT/CT diagnosing metastatic lymph nodes in esophageal squamous cell carcinoma

**DOI:** 10.1111/1759-7714.15421

**Published:** 2024-08-14

**Authors:** Xiaojin Wang, Guichao Liu, Zhanyu Li, Jiyun Shi, Mingzhu Liang, Guining Fu, Liangzhan Lv, Shaolong Ju, Yin Wang, Wenhua Xu, Fan Wang, Qingdong Cao, Hong Shan

**Affiliations:** ^1^ Department of Thoracic Surgery The Fifth Affiliated Hospital of Sun Yat‐sen University Zhuhai China; ^2^ Guangdong Provincial Engineering Research Center of Molecular Imaging The Fifth Affiliated Hospital of Sun Yat‐sen University Zhuhai China; ^3^ Department of Nuclear Medicine The Fifth Affiliated Hospital of Sun Yat‐sen University Zhuhai China; ^4^ Department of Pathology The Fifth Affiliated Hospital of Sun Yat‐Sen University Zhuhai China; ^5^ Key Laboratory of Protein and Peptide Pharmaceuticals Institute of Biophysics, Chinese Academy of Sciences Beijing China; ^6^ Department of Radiology The Fifth Affiliated Hospital of Sun Yat‐sen University Zhuhai China; ^7^ Medical Isotopes Research Center Peking University Beijing China; ^8^ Department of Interventional Medicine The Fifth Affiliated Hospital of Sun Yat‐sen University Zhuhai China

**Keywords:** ^99m^Tc‐3PRGD_2_, diagnosis, esophageal squamous cell carcinoma, lymph node metastasis, SPECT/CT

## Abstract

**Background:**

Lymph node (LN) metastasis is a significant prognostic factor for esophageal squamous cell carcinoma (ESCC), and there are no satisfactory methods for accurately predicting metastatic LNs. The present study aimed to assess the efficacy of ^99m^Tc‐3PRGD_2_ single‐photon emission computed tomography (SPECT)/computed tomography (CT) for diagnosing metastatic LNs in ESCC.

**Methods:**

A total of 15 enrolled patients with ESCC underwent ^99m^Tc‐3PRGD_2_ SPECT/CT and 18F*‐*fluorodeoxyglucose positron emission tomography‐computed tomography (^18^F‐FDG PET/CT) examinations preoperatively. High‐definition bone carving reconstruction technology (HD‐xSPECT Bone) was applied to quantitatively assess the LN's SUV_max_ via SPECT/CT. The two methods were compared for diagnosing metastatic LNs with pathology as the gold standard.

**Results:**

Among 15 patients, 23 metastatic lymph node stations (mLNSs) were predicted by SPECT/CT, with a mean SUV_max_ of 2.71 ± 1.34, of which 15 were pathologically confirmed; 32 mLNSs were predicted by PET/CT with a mean SUV_max_ of 4.41 ± 4.02, of which 17 were pathologically confirmed. The sensitivity, specificity, accuracy, positive predictive value and negative predictive value of SPECT/CT for diagnosing metastatic LNs were 62.50%, 91.30%, 85.34%, 65.22%, and 90.32%, respectively, and those of PET/CT were 70.83%, 83.70%, 81.03%, 53.13%, and 91.67%, respectively. There was no significant difference in sensitivity (*p* = 0.061) or specificity (*p* = 0.058) between the two methods. The AUC_SPECT/CT_ was 0.816 and the SUV_max_ threshold was 2.5.

**Conclusion:**

^99m^Tc‐3PRGD_2_ SPECT/CT might be an effective method for diagnosing metastatic LNs in ESCC, especially in combination with HD‐xSPECT Bone. The diagnostic efficiency of this method was noninferior to that of ^18^F‐FDG PET/CT. The SUV_max_ threshold of 2.5 showed the highest agreement with the pathology findings.

## Graphical abstract

This study utilized ^99m^Tc‐3PRGD_2_ SPET/CT imaging in combination with high‐definition bone carving reconstruction technology to propose a novel method for diagnosing metastatic lymph nodes in esophageal squamous cell carcinoma. This approach allows for the integration of targeted imaging and quantitative analysis of lesions to assess metastatic lymph nodes. The diagnostic efficiency of this method was comparable to that of ^18^F‐FDG PET/CT.

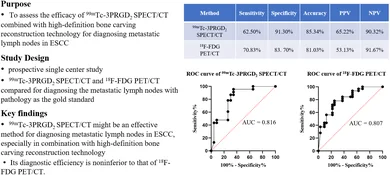

## INTRODUCTION

Esophageal cancer (EC) is one of the most common malignancies of the upper gastrointestinal tract and was responsible for 544 000 deaths every year as of 2020, accounting for 5.5% of all cancer‐related deaths.^1^ Esophageal squamous cell carcinoma (ESCC) is the most common pathological type of esophageal cancer, accounting for approximately 90% of cases in China.^2^ ESCC is not obviously symptomatic in the early stages, and approximately 64% of patients develop lymph node (LN) metastasis when diagnosed with ESCC,^3^ which is the main cause of recurrence and distant metastasis.^4^ Accurate diagnosis of metastatic LNs in ESCC is important for guiding clinical staging and making treatment decisions to improve survival time.

Molecular imaging can provide detailed images at the molecular and cellular levels, thus enabling visualization and target localization; it requires a tracer with high affinity and specificity as well as a scanner with high sensitivity and high spatial, contrast, and temporal resolutions.^5^ Positron‐emission tomography/computed tomography (PET/CT) is currently the main detection method for tumor diagnosis and staging using 18F‐fluorodeoxyglucose (^18^F‐FDG) as a tracer. However, ^18^F‐FDG is a nonspecific tracer, ^18^F‐FDG PET/CT imaging has a sensitivity of 99.4% and a specificity of 46.0%,^6^ and it cannot accurately distinguish between inflammatory and metastatic LNs.^7^ Tumor‐targeted imaging is a molecular imaging technique that uses specific molecular probes to identify and locate cancer cells. Our team has previously reported that an EGFR&c‐Met bispecific near‐infrared fluorescent probe could improve the identification of metastatic LN in a patient‐derived xenograft model of esophageal cancer.^8^ However, the long circulation time of the bispecific antibody probe limit the clinical translation for nuclear imaging. Therefore, we aimed to identify a targeted probe for the detection of metastatic lymph nodes in patients with ESCC.

Integrin α_v_β_3_ is highly expressed only in proliferating cancer cells and endothelial cells, making it a highly specific marker protein on tumor cell membranes.^9^ The Arg‐Gly‐ASP polypeptide (RGD) specifically binds to integrin α_v_β_3_. ^99m^Technetium‐three polyethylene glycol spacers‐arginine‐glycine‐aspartic polypeptide (^99m^Tc‐3PRGD_2_) has been applied as a targeted tracer for single‐photon emission computed tomography (SPECT), and multiple clinical trials have shown that it has acceptable sensitivity, specificity and safety for diagnosing lung, breast and thyroid cancers.^10^, ^11^, ^12^ Although the application of ^99m^Tc‐3PRGD_2_ SPECT imaging has been reported in esophageal disease, conventional semi‐quantitative visual analysis lacked efficiency for the diagnosis of metastatic LN in ESCC.^13^, ^14^ High‐definition bone carving reconstruction technology (HD‐xSPECT Bone) is a novel reconstruction algorithm in nuclear medicine that provides images with clear boundary delineation and improved anatomical representation of tracer activity.^15^ The combination of targeted integrin SPETCT imaging and HD‐xSPECT Bone may improve the diagnostic accuracy of metastatic LNs. In this study, we compared the diagnostic efficacy of ^99m^Tc‐3PRGD_2_ SPECT/CT with that of ^18^F‐FDG PET/CT while using pathology as the gold standard. Furthermore, we aimed to investigate whether ^99m^Tc‐3PRGD_2_ SPECT/CT could serve as an effective method for the diagnosis of metastatic LNs in ESCC when used in combination with HD‐xSPECT Bone.

## METHODS

### Patient eligibility

This prospective study was approved by the ethics committee of the Fifth Affiliated Hospital of Sun Yat‐sen University (IRB approval no. ZDWY [2020] lunzi No. K31‐1). This clinical trial (no. NCT04504565) strictly abided by the Declaration of Helsinki. Patients with ESCC who underwent surgery between September 2020 and December 2022 were enrolled, and all patients provided informed consent. The inclusion criteria were as follows: age greater than 18 years; ESCC confirmed by endoscopic biopsy pathology; and compliance with the surgical requirements through examination tests. The exclusion criteria were as follows: pregnancy or breastfeeding, no radical surgical treatment within 2 weeks after the end of the examination, weight greater than 100 kg, diabetes under poor glycemic control and withdrawal from the clinical studies halfway. All operations were performed by the same groups of surgeons.

### Procedure

**FIGURE 1:**
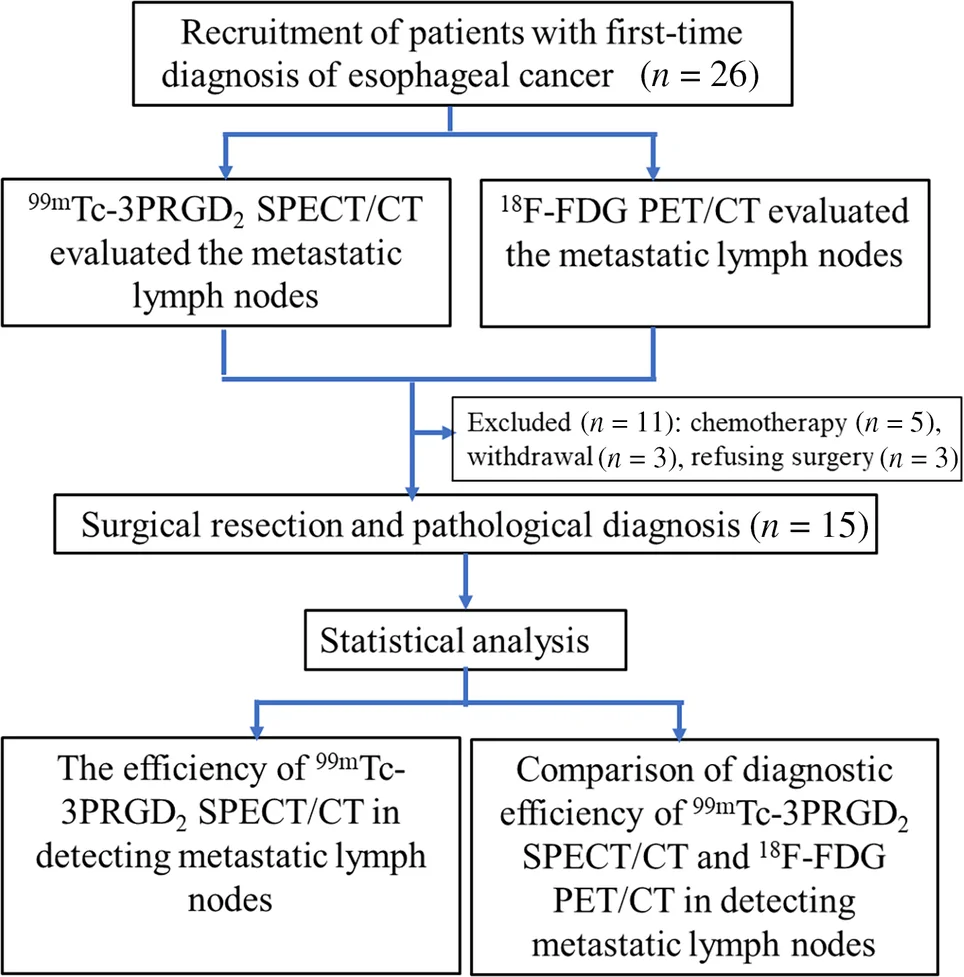
Study flow chart of the ^99m^Tc‐3PRGD_2_ single‐photon emission computed tomography (SPECT/CT) and 18F‐fluorodeoxyglucose positron emission tomography‐computed tomography (^18^F‐FDG PET/CT) for the quantitative diagnosis of metastatic lymph nodes in esophageal squamous cell carcinoma (ESCC).

The enrolled patients first underwent ^99m^Tc‐3PRGD_2_ SPECT/CT examination, followed by ^18^F‐FDG PET/CT imaging within 1 week. All images were analyzed to diagnose the primary tumor and metastatic LNs. Patients who met the surgical criteria were treated with radical surgery. After the surgery, the expression of integrin α_v_β_3_ in the excised specimens was verified by immunohistochemistry. The sensitivity, specificity, accuracy, positive predictive value (PPV), and negative predictive value (NPV) of SPECT/CT and PET/CT in the diagnosis of metastatic LNs were evaluated using postoperative pathology as the gold standard. The study flow chart is shown in Figure 1.

### SPECT/CT imaging

The 3PRGD_2_ prodrug was developed by Foshan Ruidiao Pharmaceutical Co., Ltd., and the injectable ^99m^Tc‐3PRGD_2_ was synthesized by Guangdong Xiai Pharmaceutical Co., Ltd., with a radiochemical purity exceeding 95%. ^99m^Tc‐3PRGD_2_ can be used as a tracer for SPECT imaging. The tracer was injected intravenously at a dose of 0.3 ± 0.06 mCi/kg. SPECT/CT scanning was performed approximately 45 min after intravenous injection. The images were obtained using a low‐energy/high‐resolution parallel hole collimator (matrix 256 × 256, collection time 15 min and CT parameters of 130 kV, 180 mAs, and thickness of 1.5 mm) on a Siemens Symbia Intevo Bold SPECT/CT system. In comparison with conventional SPECT imaging with lower resolution, unclear positioning, and blurred uptake, Symbia Intevo Bold SPECT/CT fusion imaging achieves precise localization of lesions. Additionally, high‐definition bone carving technology can classify high‐precision CT data into ZoneMap voxels based on xSPECT technology, and then “etch” them into large matrix SPECT images. Through OSCGM iterative reconstruction, ultra‐high definition images can be generated displaying finely detailed uptake values of lesions while providing accurate localization and quantitative analysis capabilities.

### PET/CT imaging

^18^F‐FDG was synthesized by Guangzhou HTA Pharmaceutical Co. Ltd., with a radiochemical purity greater than 95%. PET/CT scanning was performed approximately 60 min after intravenous injection at a dose of 0.15 ± 0.03 mCi/kg. Using an integrated PET/CT scanner (United Imaging uMI780) covering the top of the head to the upper thighs, PET/CT images were acquired at 3 min/bed position with the following parameters: 120 kV, 110 mAs, and thickness of 1.25 mm.

### Image diagnosis

All images were registered on the MedEx workstation. Two groups of experienced nuclear medicine physicians (two physicians in each group) independently analyzed the SPECT/CT and PET/CT images. Lymph nodes with short diameter more than 5 mm were analyzed based on CT images. Image interpretation included visual analysis and quantitative assessment. HD‐xSPECT Bone was used to measure the SUV_max_ of the focus on SPECT images. Any difference in opinion was resolved by consensus. This study classified and evaluated the lymph node station based on the location of the primary tumor of esophageal cancer (Union for International Cancer Control [UICC]/American Joint Committee on Cancer [AJCC] eighth edition, 2017).^16^ The lesions were divided into primary tumors and metastatic LNs. According to the CT images, tumor regions of interest (ROIs) were drawn around the foci with increased uptake in the trans‐axial slices. The T/B ratios and the normalized SUV_max_ (original lesion SUV_max_/mean SUV_max_ of the mediastinal blood pool) on both SPECT and PET were measured, and the average of the two groups of physicians' assessments was recorded.

### Immunohistochemistry (IHC)

IHC was performed on the pathological specimens with an anti‐α_v_β_3_ antibody (mouse monoclonal anti‐α_v_β_3_ antibody (23C6), SC‐7312, 1:150, Santa Cruz Biotechnology). Two attending pathologists independently evaluated the staining intensity and percentage of positive tumor cells.

### Statistical analysis

Statistical analyses were performed using IBM SPSS statistical software (version 22.0; SPSS, Chicago, Illinois, USA). Continuous variables related to clinical data are expressed as the median and were compared by paired *t* tests. The detection efficiency of the two methods for diagnosing metastatic LNs was compared using the paired chi‐square test. The diagnostic performance of SPECT/CT and PET/CT was compared via receiver operating characteristic (ROC) curve analysis. The mean and standard deviation were used to describe the quantitative parameters, and the relationship between the target to background ratio (T/B) and the SUV_max_ was analyzed by the Pearson correlation coefficient. A *p*‐value less than 0.05 was considered to indicate statistical significance.

## RESULTS

### Patient clinical features

This study recruited 26 patients with ESCC, five of whom opted for chemotherapy after enrollment, three of whom withdrew from the study and three of whom refused surgical treatment. A total of 15 enrolled patients (range: 39–80 years, median age: 66 years) were ultimately included in the study. The pTNM stages of 15 patients included stages IA to IVA and the proportion of stage III patients was 66.67%. The clinical characteristics of the patients are summarized in Table 1.

**TABLE 1:** Demographic and clinical characteristics of the 15 enrolled patients.

Characteristic	Data	Percentage
Age, years	61.40 ± 10.49	
Sex		
Male	12	75.00%
Female	3	25.00%
Tumor location		
Ut	3	20.00%
Mt	5	33.33%
Lt	7	46.67%
pTNM stage		
IA	1	6.67%
IB	1	6.67%
IIA	1	6.67%
IIB	1	6.67%
IIIA	5	33.33%
IIIB	5	33.33%
IVA	1	6.67%
Pathological type		
ESCC	15	100.00%

*Note*: pTNM stage was classified by the UICC/AJCC TNM classification for EC, eighth edition.^16^

Abbreviations: AJCC, American Joint Committee on Cancer; ESCC, esophageal squamous cell carcinoma; Lt, lower thoracic esophagus; Mt, middle thoracic esophagus; UICC, Union for International Cancer Control; Ut, upper thoracic esophagus.

### Performance of ^99m^Tc‐3PRGD_2_ SPECT/CT versus ^18^F‐FDG PET/CT in ESCC

**FIGURE 2:**
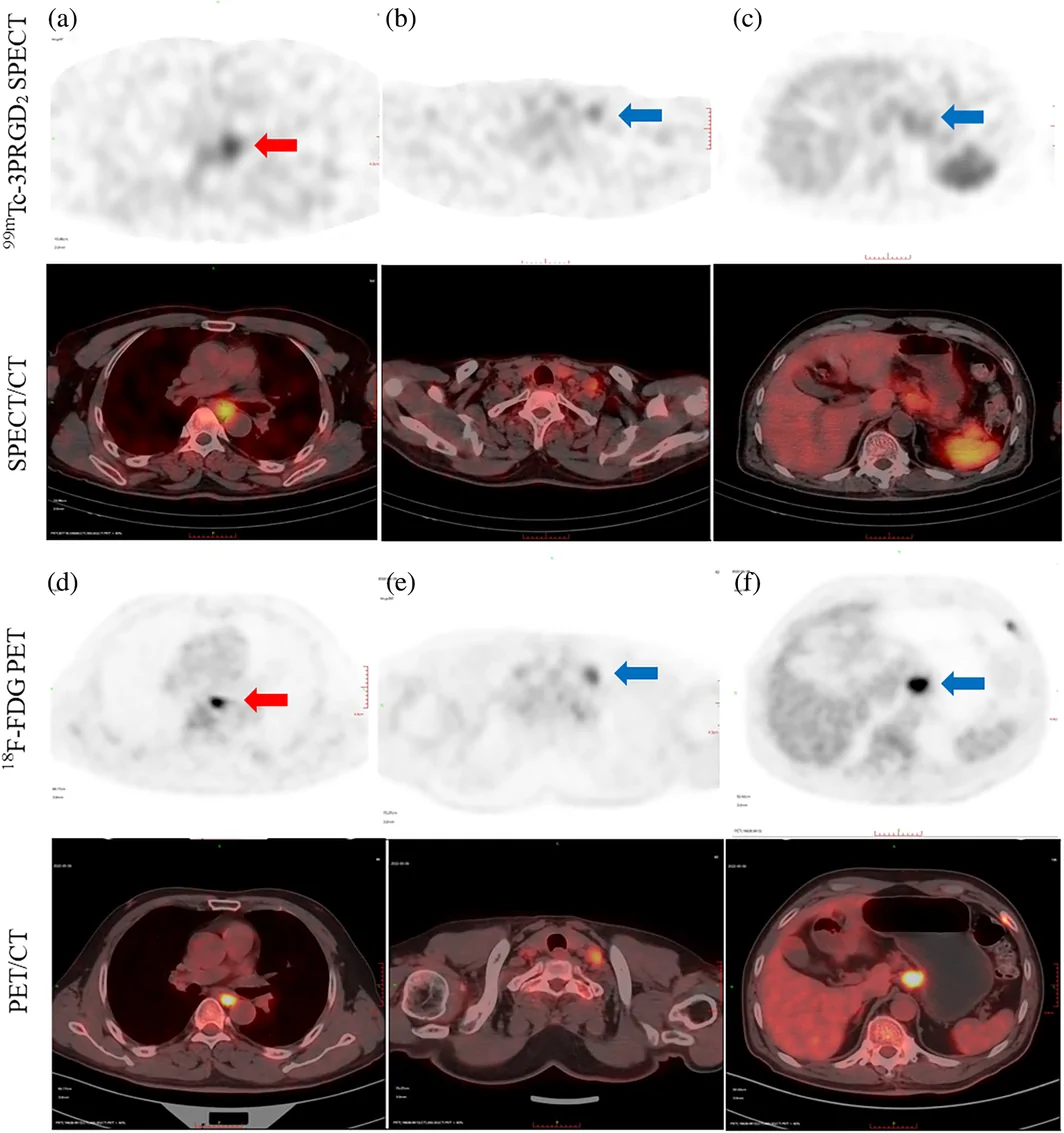
Representative images of ^99m^Tc‐3PRGD_2_ single‐photon emission computed tomography (SPECT/CT) and 18F‐fluorodeoxyglucose positron emission tomography‐computed tomography (^18^F‐FDG PET/CT). A 69‐year‐old man with esophageal squamous cell carcinoma (ESCC) and lymph node metastasis. (a) The SPECT scan demonstrating increased ^99m^Tc‐3PRGD2 uptake (SUV_max_ = 3.6) in the primary lesion. (b, c) The SPECT/CT scan demonstrating metastatic lymph nodes (No. 1L and No. 17) with increased ^99m^Tc‐3PRGD_2_ uptake (SUV_max_: 3.1; 2.9). (d) The PET scan demonstrating increased ^18^F‐FDG uptake (SUV_max_ = 3.9) in the primary lesion. (e, f) The ^18^F‐FDG PET/CT scan demonstrating metastatic lymph nodes (No. 1L and No. 17) with increased FDG uptake (SUV_max_: 6.8; 11.3). The red arrows point to the primary lesions of ESCC, and the blue arrows point to the metastatic lymph nodes.

**FIGURE 3:**
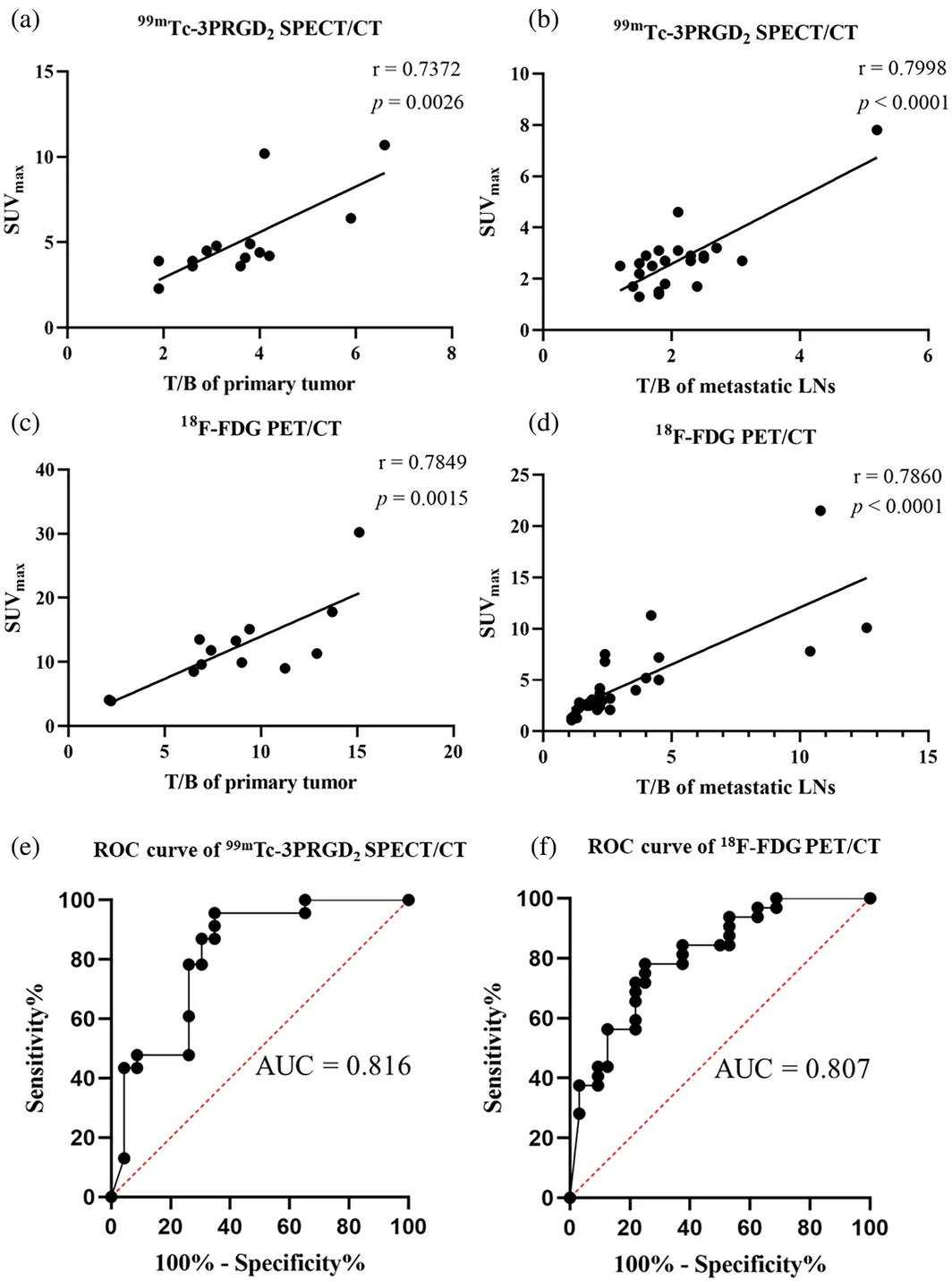
Correlation between the tumor‐to‐background ratio (T/B) value and the SUV_max_ of lesions and receiver‐operating characteristic (ROC) curves of the SUV_max_ in predicting metastatic lymph nodes by ^99m^Tc‐3PRGD_2_ single‐photon emission computed tomography (SPECT/CT) and 18F‐fluorodeoxyglucose positron emission tomography‐computed tomography (^18^F‐FDG PET/CT). (a, b) The positive correlation between the SUV_max_ and T/B value of the primary lesion and metastatic lymph nodes by ^99m^Tc‐3PRGD_2_ SPECT/CT. (c, d) The positive correlation between the SUV_max_ and T/B value of the primary lesion and metastatic lymph nodes by ^18^F‐FDG PET/CT (Pearson correlation test). (e) The receiver operating characteristic (ROC) curve of ^99m^Tc‐3PRGD_2_ SPECT/CT. (f) The ROC curve of ^18^F‐FDG PET/CT. • Indicates planar visual analysis showed no nuclide concentration in the lesions. ‐ is horizontal line, it indicates planar visual analysis showed no nuclide concentration in the lesions.

The diagnostic results obtained by nuclear medicine specialists were as follows: among 15 patients, the diagnostic rates of SPECT /CT and PET/CT for visualizing the primary tumor were both 86.67% (13/15). Two patients with stage I disease were not visualized by either SPECT/CT or PET/CT imaging. Representative ^99m^Tc‐3PRGD_2_ SPECT/CT and ^18^F‐FDG PET/CT images are shown in Figure 2. On SPECT/CT, the T/B of the primary tumor was 3.64 ± 1.40, and the SUV_max_ was 5.27 ± 2.48; 23 predicted metastatic lymph nodes stations (LNSs) were diagnosed with the T/B of 2.18 ± 0.83 and the SUV_max_ of 2.71 ± 1.34. On PET/CT, the T/B and SUV_max_ of the primary tumor were 8.61 ± 3.98 and 12.15 ± 6.70, respectively; 32 predicted metastatic LNSs were diagnosed with the T/B of 3.10 ± 2.85 and the SUV_max_ of 4.41 ± 4.02. (Table 2). The correlation coefficients of the T/B and SUV_max_ of the primary tumors and the LNSs predicted by SPECT/CT were 0.737 and 0.800, respectively (Figure 3a,b), while the correlations which the LNSs predicted by PET/CT were 0.785 and 0.786, respectively (Figure 3c,d). The ROC curves were drawn according to the SPECT/CT and PET/CT SUV_max_ of the predicted metastatic LNs. The AUC _SPECT/CT_ was 0.816, and the AUC _PET/CT_ was 0.807(Figure 3e, f). For the maximum Youden's index, the SUV_max_ threshold was 2.5 for ^99m^Tc‐3PRGD_2_ SPECT/CT and 3.0 for ^18^F‐FDG PET/CT.

**TABLE 2:** Results of ^99m^Tc‐3PRGD_2_ SPECT/CT and ^18^F‐FDG PET/CT for the diagnosis of primary lesions and mLNs.

Patient	Primary lesion T/B		Primary lesion SUV_max_		LN T/B		LN SUV_max_		Predicted mLN stations	
	SPECT	PET	SPECT	PET	SPECT	PET	SPECT	PET	SPECT	PET
1	–	–	–	–	–	–	–	–	–	–
2	3.8	12.9	4.9	11.3	1.9; 1.2; 1.9	2.6; 12.6; 10.4	1.8; 2.5; 2.7	2.1; 10.1; 7.8	1R; 2L; 2R	1L; 2L 2R
3	2.6	6.9	3.9	9.6	1.8; 2.4; 1.5	1.3; 1.2; 4.5	1.4; 1.7; 2.6	1.3; 1.5; 7.2	1R; 1L; 7	1L; 1R 16
4	6.6	11.25	10.7	9	1.8; 2.5; 2.7	2.4; 1.9; 1.8	1.5; 2.8; 3.2	7.5; 2.9; 2.5	2R; 8; 17	2R; 4R8
5	3.1	13.7	4.8	17.8	1.4; 1.7	4; 1.4	1.7; 2.5	5.2; 2.8	2R; 8	8; 17
6	2.9	9.4	4.5	15.1	3.1; 2.1	1.9; 1.6; 1.8; 1.4	2.7; 4.6	3.1; 2.62; 8; 2.3	1R; 2L	1R; 2L4; 5
7	1.9	2.2	3.9	3.9	2.1; 1.5; 2.5	2.4; 4.2	3.1; 1.3; 2.9	6.8; 11.3	2L; 7; 17	2L; 17
8	4.2	6.5	4.2	8.5	2.3	2.3; 1.1	2.7	3.0; 1.1	2R	2R; 7
9	5.9	6.8	6.4	13.5	1.4; 2.3	2.2; 1.9	1.7; 2.9	3.7; 2.7	7; 8	8; 16
10	–	–	–	–	–	–	–	–	–	–
11	4.1	15.1	10.2	30.2	1.5; 5.2	1.7; 10.8; 1.3	2.2; 7.8	2.5; 21.5; 2.1	1L; 8	2R; 8; 16
12	1.9	2.1	2.3	4.1	–	–	–	–	–	–
13	3.7	7.4	4.7	11.8	–	2.1; 2.6; 2.2	–	2.1; 3.2; 4.2	–	4; 5; 17
14	4.0	9	4.4	9.9	2.6; 2.8	4.5; 3.6; 2.2	2.9;3.1	5.0; 4.0; 2.4	2 L; 17	1L; 1R 17
15	2.6	8.7	3.6	13.3	–	1.1; 2.1	–	1.3; 2.5	–	1L; 2R
Statistical description	3.64 ± 1.40	8.61 ± 3.98	5.27 ± 2.48	12.15 ± 6.70	2.18 ± 0.83	3.10 ± 2.85	2.71 ± 1.34	4.41 ± 4.02	23	32

Abbreviations: ^18^F‐FDG PET/CT, 18F‐fluorodeoxyglucose positron emission tomography‐computed tomography; mLNs, metastatic lymph nodes; SPECT/CT, single‐photon emission computed tomography; SUV_max_, normalized maximum standardized uptake value; T/B, tumor‐to‐background ratio.

In 15 patients, a total of 364 lymph nodes were dissected and belonged to 116 LNSs. Among them, 35 metastatic LNs were pathologically diagnosed and distributed across 24 LNSs (Table 3). Of the 23 metastatic LNSs predicted by ^99m^Tc‐3PRGD_2_ SPECT/CT, 15 were confirmed, resulting in eight false positives. Among the 32 metastatic LNSs predicted by ^18^F‐FDG PET/CT, 17 were pathologically confirmed, resulting in 15 false positives. Among the 24 metastatic LNSs, ^99m^Tc‐3PRGD_2_ SPECT/CT missed 9 LNSs, and ^18^F‐FDG PET/CT missed 7 LNSs (Table 4). The sensitivity, specificity, accuracy, PPV and NPV of ^99m^Tc‐3PRGD_2_ SPECT/CT for diagnosing metastatic LNs were 62.50%, 91.30%, 85.34%, 65.22%, and 90.32%, respectively, and those of ^18^F‐FDG PET/CT were 70.83%, 83.70%, 81.03%, 53.13%, and 91.67%, respectively. There was no significant difference in sensitivity or specificity between the two methods (*p* = 0.061 and *p* = 0.058, respectively).

**TABLE 3:** Pathological diagnosis and pTNM stage of the 15 enrolled patients.

Patient	Tumor location	pTNM stage	Metastatic LNSs	Number of mLNs	Number of LNSs resected	Number of LNs resected
1	Mt	pT1bN0M0	–	0	8	24
2	Ut	pT3N2M0	2L; 2R	4	9	27
3	Ut	pT2N0M0	–	0	6	13
4	Mt	pT1N1M0	7; 16	2	6	11
5	Lt	pT3N2M0	2R; 16; 17	3	10	50
6	Mt	pT2N1M0	8	1	7	28
7	Mt	pT3N3MO	1R; 2L; 8	8	10	36
8	Ut	pT1N2M0	2L; 17	3	8	22
9	Lt	pT2N1M0	2R	1	7	23
10	Lt	pT3N1M0	8; 16	2	6	19
11	Lt	pT1aN0M0	–	0	4	6
12	Lt	pT2N2M0	2L; 2R; 8; 17	5	5	10
13	Lt	pT2N1M0	17	1	10	32
14	Mt	pT2N2M0	1L; 17	4	11	38
15	Lt	pT2N1M0	2L	1	9	25
Statistical description			24	35	116	364

Abbreviations: LNs, lymph nodes; LNSs, lymph node stations; mLNs, metastatic lymph nodes.

**TABLE 4:** Comparison between the results of ^99m^Tc‐3PRGD_2_ SPECT/CT and ^18^F‐FDG PET/CT in diagnosing mLNSs.

	Pathological mLNSs	Pathological non‐mLNSs
SPECT/CT^+^ predicted mLNSs	15 (a1)	8 (b1)
SPECT/CT^−^ predicted mLNSs	9 (c1)	84 (d1)
PET/CT^+^ predicted mLNSs	17 (a2)	15 (b2)
PET/CT^−^ predicted mLNSs	7 (c2)	77 (d2)

*Note*: Calculation formulas: sensitivity = a/(a + c); specificity = d/(b + d).

Accuracy = (a + d)/(a + b + c + d); PPV = a/(a + b); NPV = d/(c + d). Abbreviations: ^18^F‐FDG PET/CT, 18F‐fluorodeoxyglucose positron emission tomography‐computed tomography; mLNSs, metastatic lymph node stations; SPECT/CT, single‐photon emission computed tomography.

### Integrin α_v_β_3_ expression in the resected lymph nodes of ESCC

**FIGURE 4:**
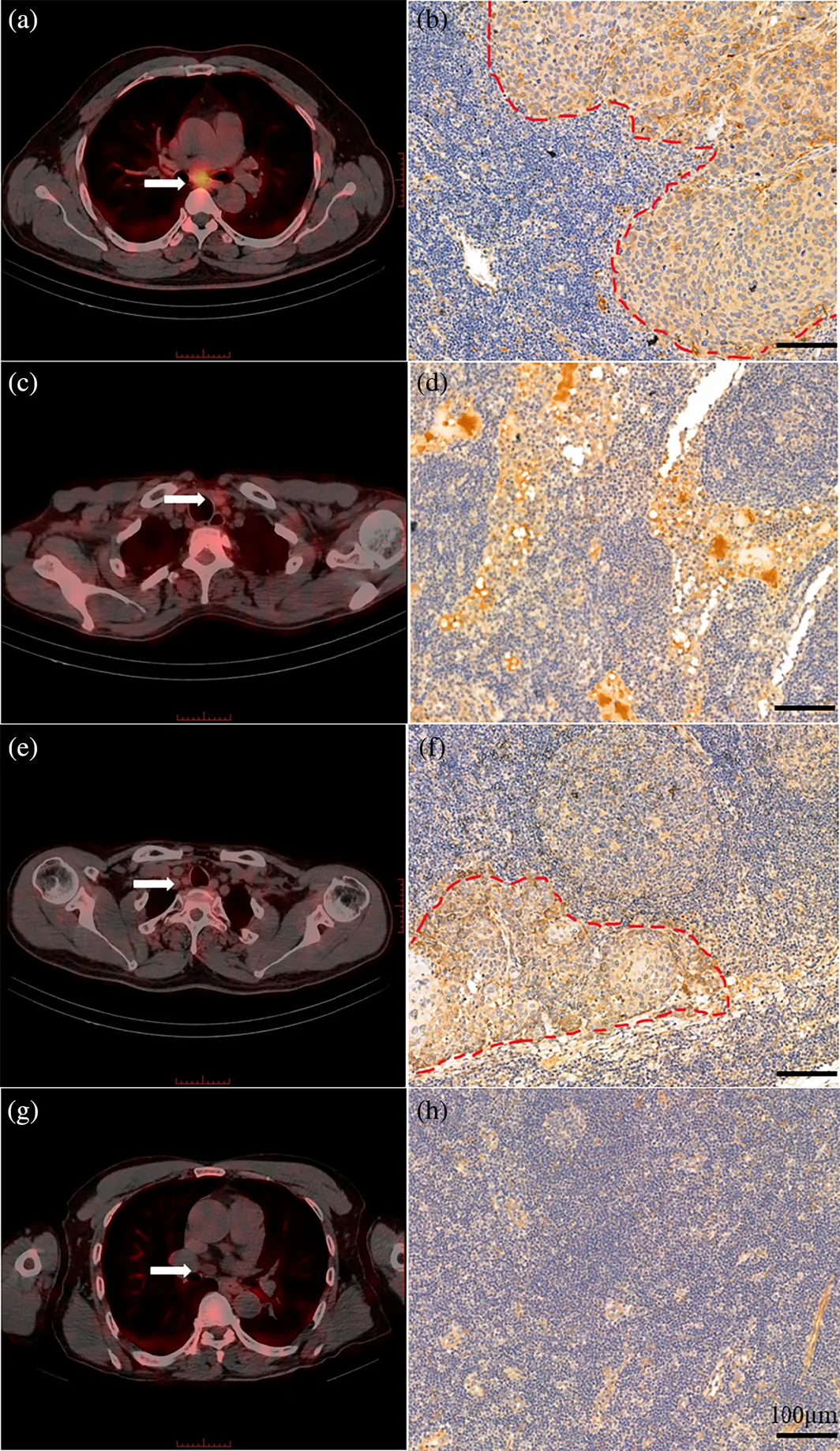
Representative lymph node (LN) images obtained with ^99m^Tc‐3PRGD_2_ single‐photon emission computed tomography (SPECT/CT) and the corresponding immunohistochemistry (IHC) for integrin α_v_β_3_. (a) Metastatic LN (SPECT^+^ pathology^+^). (b) IHC image of metastatic LN (metastatic lesion outlined with red dotted line). (c) Misdiagnosed metastatic LN (SPECT ^+^ pathology^−^). (d) IHC image of misdiagnosed metastatic LN (hyperplastic lymphoid tissue, no metastatic lesion detected). (e) Missed metastatic LN (SPECT^−^ pathology^+^). (f) IHC image of missed metastatic LN (metastatic lesion outlined with red dotted line). (g) Nonmetastatic LN (SPECT^−^ pathology^−^). (h) IHC image of nonmetastatic LN. Scale bars: 100 μm (the white arrow points to the corresponding lymph node).

A total of 77.14% (27/35) of the metastatic LNs highly expressed integrin α_v_β_3_; 86.67% (26/30) of the normal margin and nonmetastatic LNs were negative. According to ^99m^Tc‐3PRGD_2_ SPECT/CT diagnosis and pathology, the resected lymph nodes were divided into four categories: metastatic LNs (SPECT^+^ pathology^+^), missed metastatic LNs (SPECT^−^ pathology^+^), misdiagnosed metastatic LNs (SPECT ^+^ pathology^−^) and non‐metastatic LNs (SPECT^−^ pathology^−^). Representative LN images obtained with ^99m^Tc‐3PRGD_2_ SPECT/CT and corresponding IHC images of integrin α_v_β_3_ are shown in Figure 4. The expression of integrin α_v_β_3_ was strongly positive in metastatic LNs (Figure 4b) with a large metastatic lesion (6.70 mm ± 2.13 mm), scattered positive in misdiagnosed metastatic LNs (Figure 4d) with hyperplastic lymphoid tissue, weakly positive in missed metastatic LNs (Figure 4f) with a tiny metastatic lesion (1.75 mm ± 0.78 mm), and negative in nonmetastatic LNs (Figure 4h). This result indicated that the metastatic lesion volume was a key factor affecting ^99m^Tc‐3PRGD_2_ SPECT/CT imaging of metastatic LNs, the larger the metastatic lesion was, the less likely it was to be missed.

## DISCUSSION

Tumor‐targeted imaging with nuclear probes is an important method in the preclinical and clinical application of precision medicine. The binding of RGD peptides to integrin α_v_β_3_ has high specificity and affinity^17^, ^18^, ^19^ and ^99m^Tc‐3PRGD_2_ SPECT has been used in the clinical scintigraphy of lung cancer and breast cancer.^10^, ^20^ In this study, we combined ^99m^Tc‐3PRGD_2_ SPETCT/CT imaging with HD‐xSPECT Bone to propose a novel method for diagnosing metastatic lymph nodes in ESCC. For the first time, HD‐xSPECT Bone was used to quantify the SUV_max_ of LNs via ^99m^Tc‐3PRGD_2_ SPETC/CT imaging. The T/B and SUV_max_ of the predicted metastatic LNs were correlated with ^99m^Tc‐3PRGD_2_ SPECT/CT (*r* = 0.800, *p* < 0.0001). These results indicate that HD‐xSPECT Bone is a feasible method for quantifying metastatic lymph nodes via ^99m^Tc‐3PRGD_2_ SPECT/CT. In contrast, conventional SPECT images were acquired via semiquantitative (T/B) visual analysis,^13^, ^14^ lacking an effective quantitative method for diagnosing metastatic LNs. The images processed by HD‐xSPECT Bone had a higher image definition than conventional SPECT imaging, which is conducive to the accurate interpretation of small lesions and lesion margins.^21^ Additionally, this method realized the combination of targeted imaging and quantitative analysis of lesions to assess metastatic LNs.

This study evaluated the value of ^99m^Tc‐3PRGD_2_ SPECT/CT imaging for diagnosing the metastatic LNs and compared it with that of ^18^F‐FDG PET/CT. There was no significant difference in sensitivity (62.50% vs. 70.83%) or specificity (91.21% vs. 83.52%) between the two methods (*p* = 0.061 and *p* = 0.058, respectively). These results indicate that ^99m^Tc‐3PRGD_2_ SPECT/CT is an effective targeted imaging method for the diagnosis of metastatic LNs in ESCC, based on the limited number of patients.

In animal models of lung cancer, the specificity and accuracy of ^99m^Tc‐3PRGD_2_ SPECT/CT were better than those of ^18^F‐FDG PET/CT.^22^ In this study, the accuracy of ^99m^Tc‐3PRGD_2_ SPECT/CT (85.34%) was slightly higher than that of ^18^F‐FDG PET/CT (81.03%) for diagnosing metastatic LNs. According to the ROC curve analysis, the AUC for ^99m^Tc‐3PRGD_2_ SPECT/CT (0.816) was approximately the same as the AUC for ^18^F‐FDG PET/CT (0.807). The above results show that the diagnostic performance of the two techniques is comparable. In terms of diagnostic sensitivity and specificity, ^99m^Tc‐3PRGD_2_ SPECT/CT was noninferior to ^18^F‐FDG PET/CT for the diagnosis of metastatic lymph nodes in ESCC when using pathology as the gold standard. Encouragingly, ^99m^Tc‐3PRGD_2_ SPECT/CT has initiated the clinical application of targeted imaging for metastatic LNs in ESCC.

In this study, there were eight misdiagnosed metastatic LNs (SPECT ^+^ pathology^−^) in ^99m^Tc‐3PRGD_2_ SPECT/CT imaging. Immunohistochemical and hematoxylin–eosin staining were performed on these lymph nodes, revealing that the hyperplastic lymphoid tissue in these lymph nodes was rich in capillaries, which increased the binding with ^99m^Tc‐3PRGD_2_ and led to false positive lymph nodes. Additionally, there were nine missed metastatic lymph nodes with tiny lesions (lesion diameter 1.75 mm ± 0.78 mm). It is speculated that the small size of these lesions resulted in a weak signal for ^99m^Tc‐3PRGD_2_ targeted imaging, resulting in lower sensitivity.

There were several limitations in this study. As a prospective study, the number of patients enrolled is currently small, and further multicenter studies with larger amounts of sample sizes are needed to validate our results. Due to the insufficient spatial resolution of SPECT images and the strong correlation between nuclide uptake and tumor volume, ^99m^Tc‐3PRGD_2_ SPECT/CT can be used to diagnose metastatic lymph nodes only at the lymph node station, and cannot accurately determine the number of metastatic lymph nodes.

In conclusion, ^99m^Tc‐3PRGD_2_ SPECT/CT might be an effective targeted imaging method for the diagnosis of metastatic LNs in ESCC, especially in combination with HD‐xSPECT Bone. The diagnostic efficiency of this method was noninferior to that of ^18^F‐FDG PET/CT. When the SUV_max_ cutoff value was 2.5, the imaging modality had the highest agreement with the pathology findings.

## AUTHOR CONTRIBUTIONS

**Xiaojin Wang**: Conceptualization, Writing ‐ Original Draft; **Guichao Liu**: Formal analysis; **Zhanyu Li**: Visualization; **Jiyun Shi**: Methodology; **Mingzhu Liang**: Validation, Funding acquisition; **Guining Fu**: Investigation; **Liangzhan Lv & Shaolong Ju**: Data Curation; **Yin Wang**: Validation; **Wenhua Xu**: Resources; **Fan Wang**: Supervision; **Qingdong Cao**: Writing ‐ Review & Editing, and **Hong Shan**: Conceptualization, Funding acquisition.

## CONFLICT OF INTEREST STATEMENT

Xiaojin Wang, Guichao Liu, Zhanyu Li, Jiyun Shi, Mingzhu Liang, Guining Fu, Liangzhan Lv, Shaolong Ju, Yin Wang, Wenhua Xu, Fan Wang, Qingdong Cao, and Hong Shan have no relevant financial or non‐financial conflicts of interest to disclose for the article.

## Data Availability

The data that support the findings of this study are available from the corresponding author upon reasonable request. The data are not publicly available due to privacy or ethical restrictions.
